# Crystal structure of the dog allergen Can f 6 and structure-based implications of its cross-reactivity with the cat allergen Fel d 4

**DOI:** 10.1038/s41598-018-38134-w

**Published:** 2019-02-06

**Authors:** Kenji Yamamoto, Osamu Ishibashi, Keisuke Sugiura, Miki Ubatani, Masaya Sakaguchi, Masatoshi Nakatsuji, Shigeru Shimamoto, Masanori Noda, Susumu Uchiyama, Yuma Fukutomi, Shigenori Nishimura, Takashi Inui

**Affiliations:** 1 Department of Applied Life Sciences, Graduate School of Life and Environmental Sciences, Osaka Prefecture University, 1-1 Gakuen-cho, Naka-ku, Sakai, 599-8531 Japan; 2 Faculty of science and engineering, Kinki University, 3-4-1 Kowakae, Higashi-Osaka, 577-8502 Japan; 3 Department of Biotechnology, Graduate School of Engineering, Osaka University, 2-1 Yamadaoka, Suita, 565-0871 Japan; 4 Clinical Research Center for Allergy and Rheumatology, Sagamihara National Hospital, 18-1 Sakuradai, Minami-ku, Sagamihara, 252-0392 Japan

## Abstract

Several dog allergens cause allergic reactions in humans worldwide. Seven distinct dog allergens, designated *Canis familiaris* allergen 1 to 7 (Can f 1–Can f 7), have been identified thus far. Can f 6 shows high sequence similarity and cross-reactivity with Fel d 4 and Equ c 1, major cat and horse allergens, respectively. This study was conducted on the allergenic epitopes of Can f 6 based on its structural characterization. We demonstrated that sera from 18 out of 38 (47%) dog-sensitized patients reacted to recombinant Can f 6 protein (rCan f 6). We then determined the crystal structure of rCan f 6 by X-ray crystallography, which exhibited a conserved tertiary structural architecture found in lipocalin family proteins. Based on the tertiary structure and sequence similarities with Fel d 4 and Equ c 1, we predicted three IgE-recognizing sites that are possibly involved in cross-reactivity. Substituting three successive amino acids in these sites to triple alanine decreased IgE reactivity to the allergen. However, the degree of reduction in IgE reactivity largely depended on the site mutated and the serum used, suggesting that Can f 6 is a polyvalent allergen containing multiple epitopes and Can f 6-reactive sera contain varied amounts of IgE recognising individual Can f 6 epitopes including those predicted in this study. We also demonstrated that the predicted epitopes are partly involved in IgE cross-reactivity to Fel d 4. Interestingly, the effect of the mutation depended on whether the protein was structured or denatured, indicating that the bona fide tertiary structure of Can f 6 is essential in determining its IgE epitopes.

## Introduction

Exposure to pet-derived allergens is a major risk factor for allergy development^1^. In particular, the domestic dog, *Canis familiaris*, is a significant source of indoor allergens, found in its saliva, dander, sweat, and urine, that cause allergy symptoms such as asthma and allergic rhinitis. Due to an increase in indoor breeding leading to frequent and intimate contact between dogs and humans, dog allergies have become increasingly prominent worldwide, especially in advanced nations^2–4^.

Seven proteins designated serially as Can f 1 to Can f 7 have been identified as dog allergens. Can f 1, Can f 2, Can f 4, and Can f 6 are members of the lipocalin family, which is composed of various secretory lipid-transport proteins^5–7^. On the other hand, Can f 3, Can f 5, and Can f 7 have been classified as serum albumin, urinary kallikrein, and epididymal secretory protein E1/Niemann pick type C2 protein, respectively^8–10^. In allergology, lipocalins represent a significant protein group as major mammalian respiratory allergens from dogs, horses, cats, rats, and mice belong to this family^11,12^. Lipocalins are characterised by a common tertiary structure exhibiting an eight-stranded, antiparallel β-sheet closed back on itself forming a continuous hydrogen-bonded β-barrel^12^. The tertiary structures of Can f 2 and Can f 4 have been previously determined^13,14^, while those of Can f 1 and Can f 6 have not. Of the lipocalin allergens, Can f 1 was reported as a major dog allergen, where no less than 50–75% of dog-allergic subjects were sensitised^5,12^. Can f 6, a recently identified lipocalin allergen produced from the submaxillary gland and detected in dander^7^, shows a high amino acid sequence identity with Fel d 4 (67%) and Equ c 1 (55%), major cat and horse lipocalin allergens, respectively^15^. Consistent with this finding, sera from dog-sensitized patients with immunoglobulin E (IgE) to Can f 6 show cross-reactivity with Equ c 1 and Fel d 4^15,16^. It has been reported that 38% of dog- and cat-sensitised patients possess Can f 6-reactive IgE^15^. These reports suggested that Can f 6 and its homologous allergens possibly contribute to multi-sensitization, leading to allergic symptoms commonly caused by contact with different mammals.

In this study, we produced a recombinant protein of the dog lipocalin allergen Can f 6 (rCan f 6) and determined its three-dimensional structure at 2.35 Å resolution. We then predicted the IgE epitopes of Can f 6 *in silico* based on its structural information and the presence of charged residues, a frequent feature of epitopes identified previously. Moreover, we demonstrated the validity of the prediction using mutated rCan f 6 proteins generated by site-directed mutagenesis.

## Results

### Production and purification of rCan f 6

**Figure 1:**
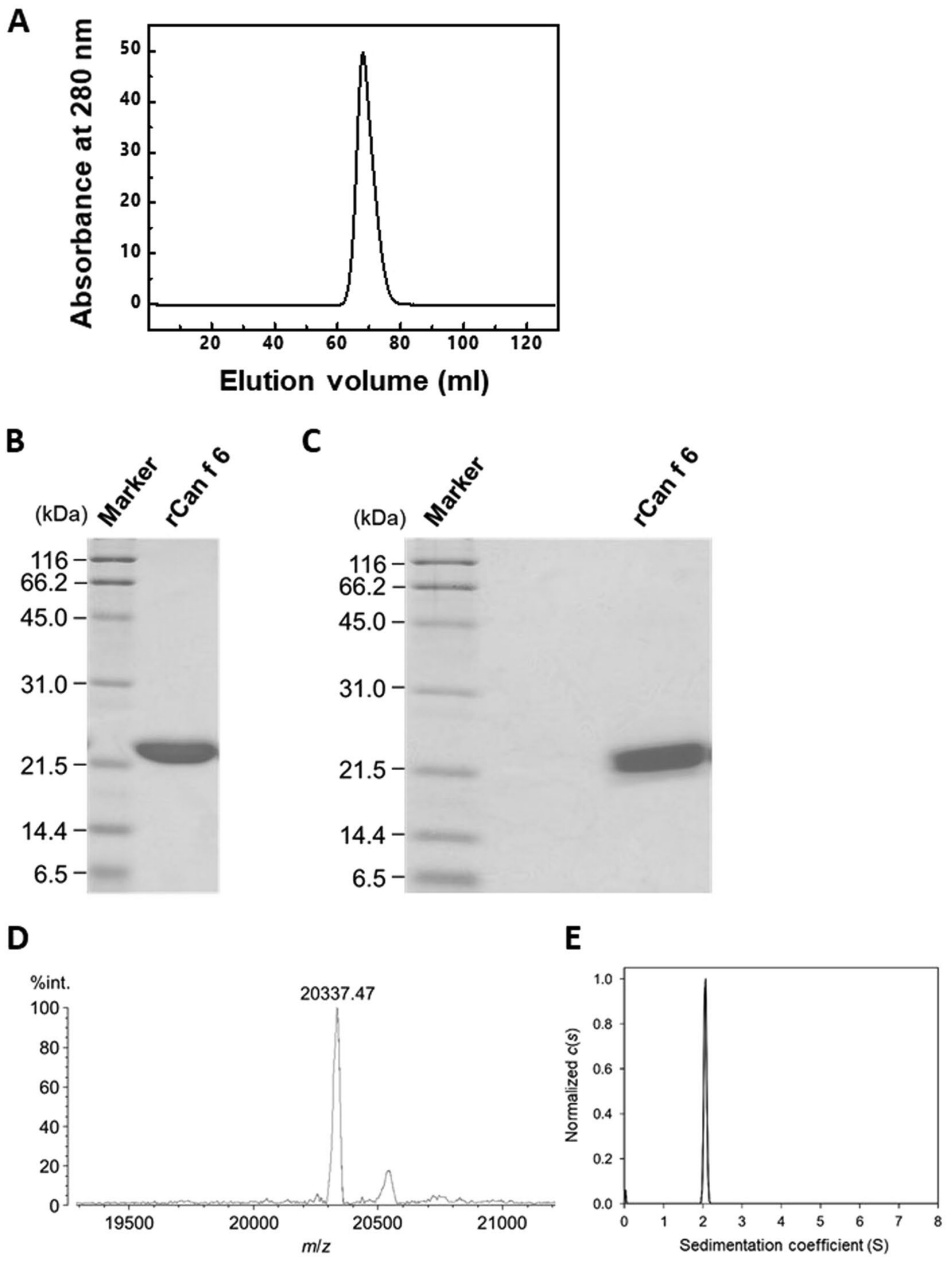
Purification of rCan f 6. (**A**) Gel filtration chromatogram of the purified rCan f 6. (**B**,**C**) SDS-PAGE profiles of rCan f 6. Purified recombinant protein (3 µg/lane) was electrophoretically separated under (**B**) reducing or (**C**) non-reducing conditions and then stained with Coomassie Brilliant blue. (**D**) Matrix assisted laser desorption/ionization-time of flight (MALDI-TOF) mass spectra of rCan f 6. Mass spectrometry of the purified recombinant protein was carried out in the linear mode using sinapinic acid as a matrix. The *m/z* value of the main peak (20337.47) corresponds to the deduced molecular mass of the recombinant protein. The sub-peak (*m/z* 20550) is considered to be derived from rCan f 6 complexed with sinapinic acid. (**E**) Distribution states of rCan f 6 analysed by AUC-SV. The molecular mass of rCan f 6 was calculated as 19.9 kDa.

Purified rCan f 6 yield was calculated to be 21 mg/L using the absorbance at 280 nm. Gel filtration chromatogram of rCan f 6 exhibited a single peak (Fig. 1A), indicating successful purification. The purity of rCan f 6 was also verified by SDS-PAGE which yielded a single band. Under reducing conditions, rCan f 6 migrated to the approximate position of 22 kDa (Fig. 1B), which corresponds to the theoretical molecular mass deduced from its amino acid sequence. A similar migration pattern was also observed under non-reducing conditions (Fig. 1C), indicating that four cysteine residues of rCan f 6 (Cys67, Cys74, Cys141, and Cys160) do not form intermolecular disulphide bonds. Furthermore, mass spectrometry revealed that the molecular mass of rCan f 6 is 20337.47, which is almost identical to the mass deduced from its amino acid sequence (20336.94; Fig. 1D).

To investigate the assembly state of rCan f 6 in solution, we performed analytical ultracentrifugation-sedimentation velocity (AUC-SV), which provides the molecular mass of a protein or association state for reversibly-interacting proteins in solution. The majority (more than 90%) of rCan f 6 species had a sedimentation value (s-value) of 2.0 S (Fig. 1E). The estimated molecular mass was 19.9 kDa, indicating that rCan f 6 is present as a monomer in solution.

### IgE binding capacity to rCan f 6

**Figure 2:**
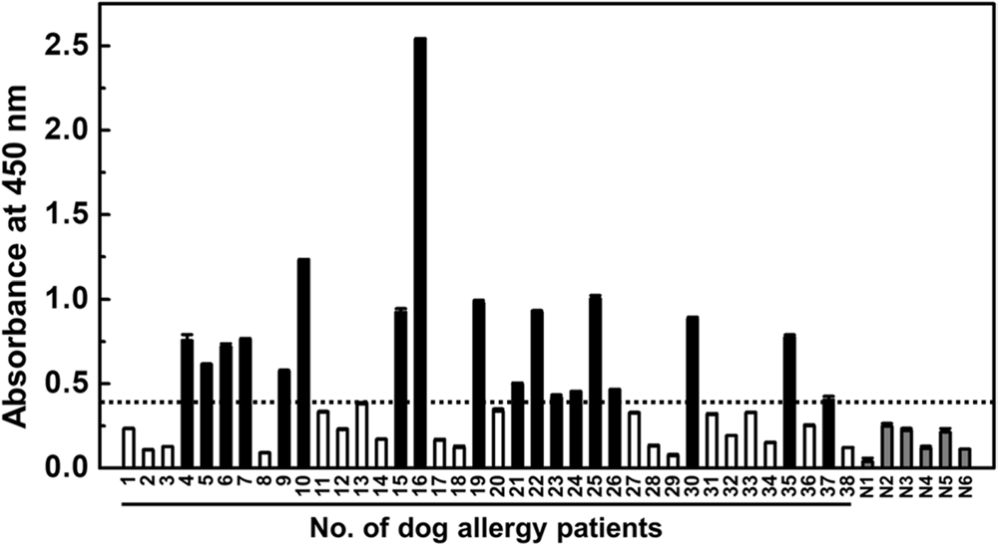
Can f 6-specific IgE levels in sera of dog-allergic patients. Sera from 18 out of 38 (47%) dog-allergic patients show Can f 6-reactive IgE levels above the cut-off value (the mean of non-dog-allergic subjects +3× SD), as indicated by black bars. White bars indicate sera from 20 dog-allergic patients that show Can f 6-reactive IgE levels below the cut-off value. Sera from 6 non-allergic donors are indicated by N1 to N6 (grey bars). The data are expressed as the mean ± SD (n = 3).

rCan f 6-specific IgE levels in sera from 38 dog-allergic patients were evaluated by direct ELISA (Fig. 2). Eighteen out of 38 (47%) sera samples were shown to react with rCan f 6. In particular, reactivity of the serum from patient 16 was highly pronounced compared with other sera. The reactivity of other sera samples was below the cut off value (mean of non-dog-allergic donors +3× standard deviation [SD]), as described previously^17^. It was also demonstrated by western blotting that rCan f 6 reacts with IgE in the serum from patient 16, but not sera from the 6 non-dog-allergic donors (Supplementary Fig. S1).

### Crystal structure of Can f 6

rCan f 6 crystals diffracted to 2.35 Å resolution and the structure was refined to an R-factor of 20.3% and an R_free_ of 26.9%. The Ramachandran plot for the rCan f 6 model indicated that 94.9% of the residues were in the preferred regions, while 4.5% of the residues were in allowed regions. Due to unclear electron density, Asp80 and Asp102 residues of chain B, Asn37 of chain C, and Lys79 of chain D were found in the disallowed regions. Asp6–Arg164 of chain A, Asp6–Ser166 of chain B, Asn5–Arg164 of chain C, and Asp6–Gln162 of chain D were the constructed regions. The final structure and refinement statistics are shown in Table 1.

**Table 1:** Data collection and refinement statistics.

Data collection	
Sample	Can f 6
Resolution range (Å)	50.00–2.35 (2.39–2.35)
Space group	P4_1_
**Cell dimensions (Å, °)**	
a = b	96.60
c	85.60
α = β = γ	90.0
Completeness (%) (overall/most outer shell)	100/100
R_merge_ (%) (overall/most outer shell)	9.7/45.9
Number of molecules per asymmetric unit	4
Number of observed reflections	448708
Number of unique reflections	32931
Multiplicity (overall/most outer shell)	13.6/14.3
I/σ(I) (overall/most outer shell)	52.2/8.11
**Refinement**	
No. of protein atoms	5008
No. of amino acid residues	637
No. of water molecules	75
No. of diethylene glycol	4
R_factor_ (%)	20.3
R_free_ (%)	26.9
**Ramachandran plot (%)**	
Preferred regions	94.9
Allowed regions	4.5
Outliners	0.6
PDB ID	5 × 7Y

For the final model, four rCan f 6 molecules consisting of chains A to D were contained in an asymmetric unit and had nearly identical structures with an average root mean square deviation (RMSD) of 0.63 Å for main-chain atoms. Supplementary Fig. S2 shows the average RMSD of main-chain atoms from the mean structure averaged over the four molecules in the asymmetric unit as a function of the residue number. Two regions with high RMSD, Leu50–Ser53 and Val111–Gln115, are located on the extruded turn and exposed to the solvent (Supplementary Fig. S2). For Leu50–Ser53, chain C exhibits an alternate conformation due to the lack of hydrogen bond(s) between the Oδ/Nδ atom(s) of Asn52 and Oγ atom of Ser54. The high RMSD at Val111–Gln115 reflects differences in packing environments among the four molecules. Thus, the residues in these regions are more flexible and may exhibit conformational variations. All other regions are superimposable among the four molecules in the asymmetric unit with low RMSD, indicating that the four molecules have essentially the same structures. Therefore, the structure of chain A is described as a representative of the four molecules.

**Figure 3:**
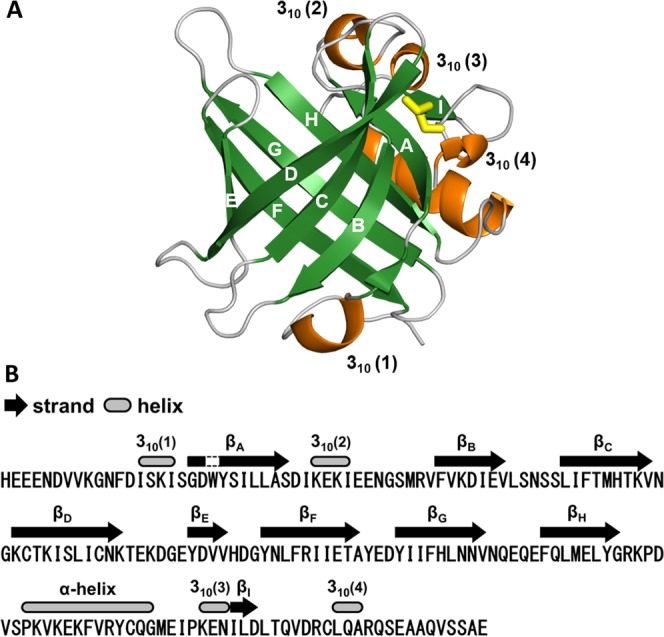
X-ray crystal structure of Can f 6 with characteristics typical of lipocalin-like proteins. (**A**) Tertiary structure of rCan f 6 (chain A) represented in a ribbon diagram. A β-barrel structure composed of 8 β-strands (indicated by A–H) is shown in green, while an α-helix and 3_10_-helix structures are indicated in orange. Yellow sticks represent an intramolecular disulfide bond. (**B**) Positions of typical secondary structures in rCan f 6 (chain A) are represented along with its amino acid sequence.

Figure 3A illustrates the overall structures of rCan f 6. The three-dimensional structure of Can f 6 possesses the conserved structural architecture of lipocalin family proteins^17,18^, an eight-stranded antiparallel β-barrel (A, Gly19–Ser28; B, Phe43–Val49; C, Leu55–Val63; D, Lys66–Lys76; E, Tyr83–Val86; F, Tyr90–Ala99; G, Tyr103–Val111; H, Phe117–Gly124) interrupted by β-hairpin-forming loops and an accessory α-helix (Pro131–Gly143; Fig. 3B). The Cys67 and Cys160 residues form an intramolecular disulphide bond, which is also highly conserved among lipocalins.

### IgE epitope prediction

**Figure 4:**
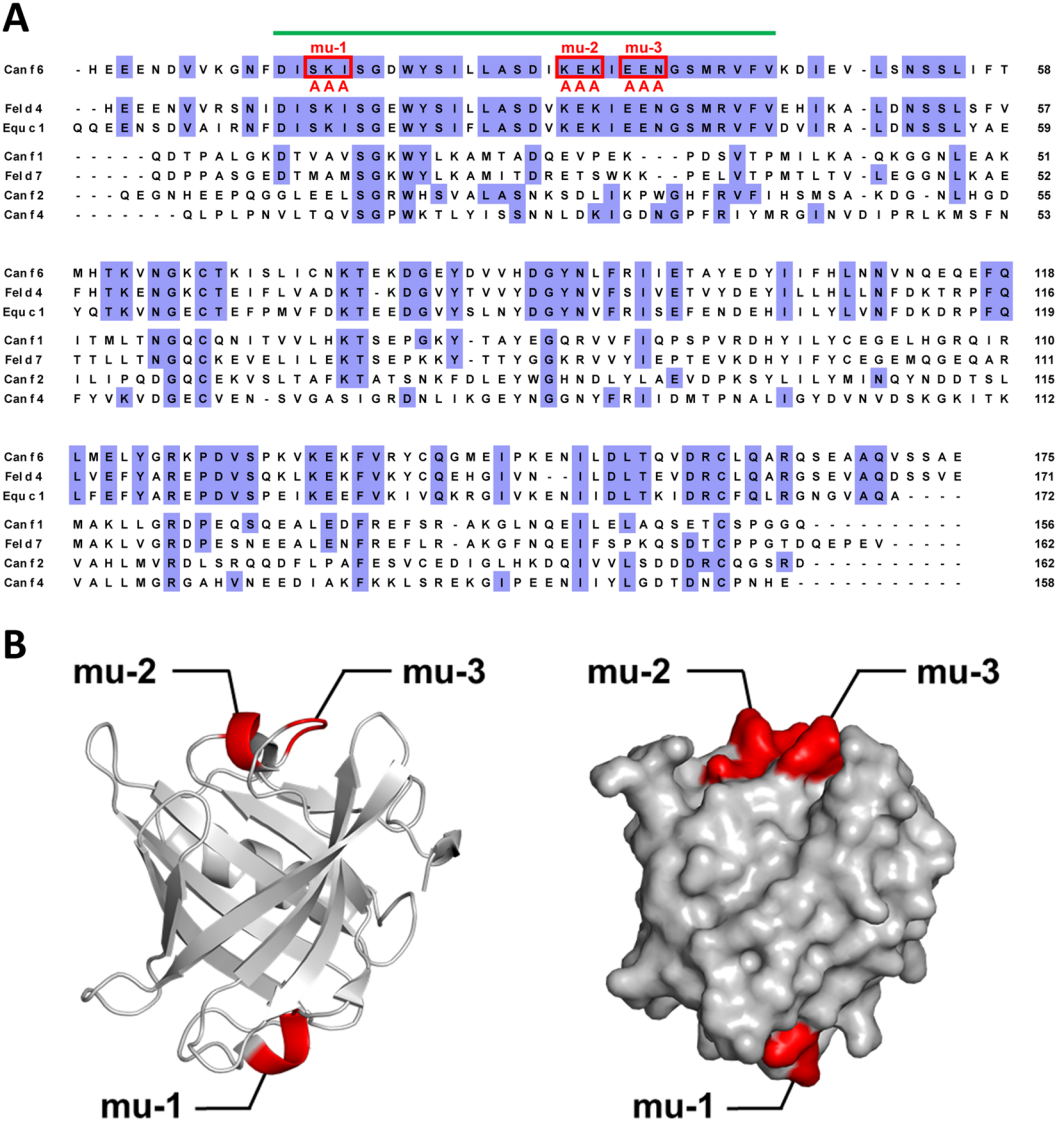
Introduction of mutations in predicted Can f 6 epitopes. (**A**) Highly conserved region among Can f 6, Fel d 4, and Equ c 1—but not among other representative lipocalin allergens (indicated by a green bar above the sequences)—was predicted to contain IgE epitope(s) involved in cross-reactivity. Three sites composed of three successive amino acids containing charged residues were substituted with triple alanine, and were designated as rCan f 6-mu-1, mu-2, and mu-3. (**B**) Schematic representation of the mutation sites (shown in red) in a ribbon diagram (left) and surface model (right) of rCan f 6.

According to previous reports of Can f 6, Fel d 4, and Equ c 1 showing IgE cross-reactivity^14^, it is highly possible that these allergens share common epitopes. Thus, we hypothesised that a surface-facing region with highly conserved amino acid sequences among the three allergens may contain epitopes. Since the importance of charged residues in IgE epitopes was previously suggested^19–21^, three successive amino acids containing charged residue(s) were substituted with triple alanine (Fig. 4A) to generate three mutated proteins designated rCan f 6-mu-1, mu-2, and mu-3. SDS-PAGE migration patterns of the purified mutated proteins under reducing conditions were consistent with the molecular mass calculated from their amino acid sequences (Supplementary Fig. S3A). SDS-PAGE under non-reducing conditions revealed that these mutated proteins, as well as rCan f 6, lack an intermolecular disulphide bond (Supplementary Fig. S3B). The size of the mutated rCan f 6 proteins was also verified by mass spectrometry, whose molecular mass nearly corresponds with their amino acid sequences (Supplementary Fig. S4A). AUC-SV experiments revealed that all mutated proteins are monomeric in solution (Supplementary Fig. S4B). Furthermore, circular dichroism spectroscopy analysis demonstrated that the mutation did not affect the secondary structure of rCan f 6 (Supplementary Fig. S5).

**Figure 5:**
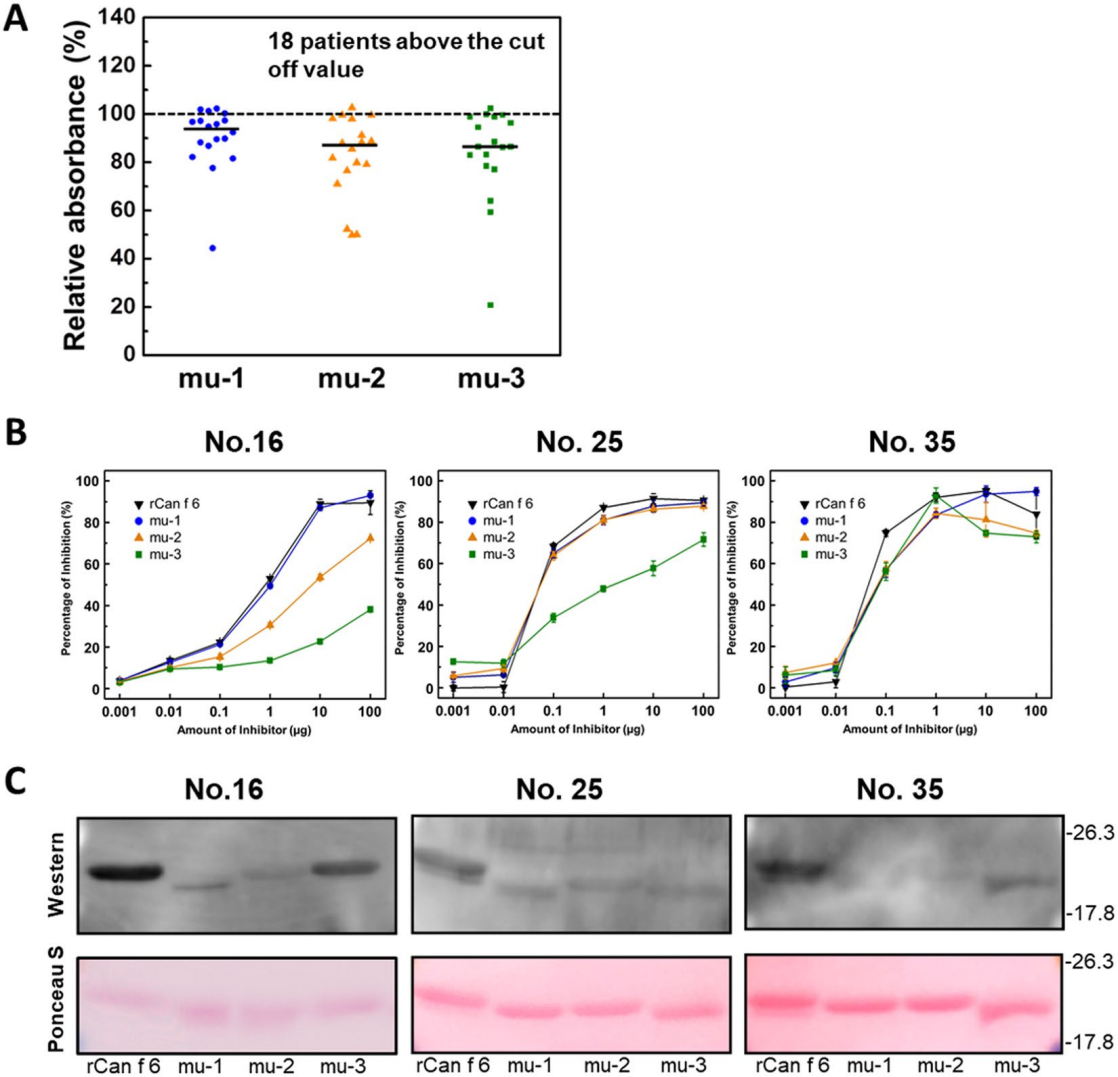
Effect of predicted Can f 6 epitope mutations on IgE reactivity. (**A**) Relative IgE reactivity to the mutated rCan f 6 proteins compared with rCan f 6 was evaluated by ELISA. Can f 6-reactive sera from 18 patients were subjected to this assay. Lines in individual columns denote the median. (**B**) Inhibition ELISA to assess specific IgE binding to immobilized rCan f 6 in the presense of wild-type and mutated rCan f 6 proteins as competitors. Sera from patients 16, 25 and 35 were used for this assay. (**C**) IgE-western blotting of Can f 6. rCan f 6 and mutated rCan f 6 proteins were subjected to SDS-PAGE and then transferred to PVDF membranes. The blots were treated with the serum from the 3 patients (upper panels). The blots were also stained with Ponceau S to verify proper protein transfer (lower panels).

The IgE-reactivity of rCan f 6-mu-1, mu-2, and mu-3 was tested using rCan f 6-reactive sera from 18 patients (Fig. 2) by ELISA. As shown in Fig. 5A, IgE-reactivity with rCan f 6-mu-2 and mu-3 was reduced by approximately 15% compared with rCan f 6, while IgE-reactivity with rCan f 6-mu-1 was reduced only by approximately 7% (Fig. 5A). We then performed inhibition ELISA experiments using Can f 6-reactive sera from 3 patients (Fig. 5B). Regardless of the serum used, IgE binding to immobilized rCan f 6 was competitively inhibited by rCan f 6-mu-1 as well as by rCan f 6, indicating that the mutated site of rCan f 6-mu-1 was not recognized as an epitope of IgE in these sera. The effects of mutations in rCan f 6-mu-2 and -mu-3 on IgE-Can f 6 binding were dependent on the serum used. When serum from patient no. 16 was used, IgE-Can f 6 binding was inhibited less by rCan f 6-mu-2 and rCan f 6-mu-3 compared to that by rCan f 6. When serum from patient no. 25 was used, IgE-Can f 6 binding was inhibited less by rCan f 6-mu-3 than rCan f 6, while mutation in Can f 6-mu-2 had no effect. By contrast, when serum from patient no. 35 was used, none of the mutations in Can f 6-mu-1, -mu-2, and -mu-3 had an effect on IgE-Can f 6 binding. As control, mutations were also introduced at two sites within surface-facing regions that are less conserved among the lipocalin allergens to generate rCan f 6-cont-1 and cont-2 proteins (Supplementary Fig. S6A,B). ELISA revealed that IgE reactivity with Can f 6 was not affected by these mutations (Supplementary Fig. S6C). Interestingly, western blotting under an SDS-PAGE condition revealed the different effects of mutation on binding of serum IgE to Can f 6 (Fig. 5C). When serum from patient no. 25 was used, all mutated rCan f 6 proteins showed a pronounced reduction in IgE reactivity compared to that of rCan f 6. When sera from patients no. 16 and no. 35 were used, rCan f 6-mu-1 and -mu-2 showed a pronounced reduction in IgE binding as well, but rCan f 6-mu-3 showed only a slight reduction in IgE binding. These results suggest that Can f 6 proteins in native and denatured (unstructured) forms exhibit different IgE reactivities.

### Evaluation of IgE cross-reactivity between Can f 6 and Fel d 4 by inhibition ELISA

**Figure 6:**
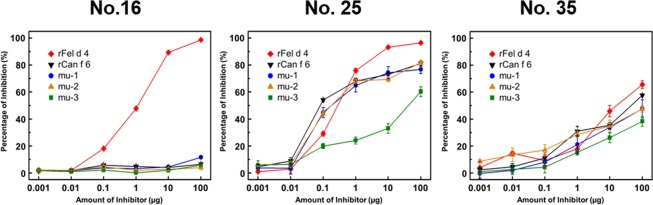
Analysis of IgE cross-reactivity between Can f 6 and Fel d 4. Inhibition ELISA to assess specific IgE binding to immobilized rFel d 4 in the presence of rFel d 4, wild-type and mutated rCan f 6 proteins as competitors. Sera from patients 16, 25 and 35 were used for this assay.

We also performed inhibition ELISA experiments to assess the binding of serum IgE to recombinant Fel d 4 (rFel d 4) (Fig. 6). When sera from patients no. 25 and no. 35 were used, IgE-rFel d 4 binding was competitively inhibited by rCan f 6, consistent with cross-reactivity between these allergens. Further, the effects of rCan f 6 mutation on IgE-rCan f 6 binding (Fig. 5B) and IgE-rFel d 4 binding were similar. To our surprise, when serum from patient no. 16 was used, IgE-rFel d 4 binding was not inhibited by wild-type or mutated rCan f 6 proteins, while such binding was fully inhibited by rFel d 4 as expected. This result indicates that this serum contains IgE recognizing Can f 6 or Fel d 4 specifically but not IgE cross-reacting with them.

## Discussion

To the best of our knowledge, this is the first report describing the crystal structure of the dog lipocalin allergen Can f 6. Using structural information, we determined the Can f 6 epitopes possibly involved in cross-reactivity with Fel d 4 and Equ c 1, cat and horse lipocalin allergens, respectively. Since accumulating evidence suggests that conformational epitopes are important in IgE cross-reactivity^22^, it is conceivable that structured regions of allergens are necessary for accurate epitope determination.

A previous study demonstrated that sera from 38% of dog-allergic patients possessed Can f 6-reactive IgE^15^. Although the rate of Can f 6-reactive sera was slightly higher in the present study (47%), this difference can be explained by the cross-reactivity between Can f 6 and Fel d 4 that was demonstrated previously^15^. The possibility that Fel d 4-sensitised cat-allergic patients were counted as dog-allergic patients, or vice versa, complicates the accurate diagnosis of pet allergies. This is supported by a previous study showing that 61% of sera from a mixture of cat- and dog-exposed allergic patients are reactive to Can f 6^7^.

The crystal structures of Can f 2, Can f 4, and Equ c 1 have already been determined^13,14,23^. Hilger *et al*.^7^ modelled the tertiary structure of Can f 6 and Fel d 4 *in silico* based on the crystal structure of Equ c 1 and showed that these allergens share highly conserved structural characteristics as well as amino acid sequence identity. Consistent with these findings, the X-ray crystal structure characteristics of Can f 6 remarkably resembled those of Equ c 1 (Supplementary Fig. S7A). Interestingly, Can f 2 and Can f 4, which share relatively low amino acid sequence identities with Can f 6 (Fig. 4A), also exhibited high similarities with Can f 6 in terms of their X-ray crystal structures (Supplementary Fig. S7B). However, the cross-reactivities of these allergens to Can f 6, Fel d 4, or Equ c 1 have not been reported. Thus, both primary and tertiary structures are considered important for IgE-epitope binding.

The results of our ELISA experiments suggest that Can f 6 is a polyvalent allergen containing multiple epitopes and Can f 6-reactive sera contain varied amounts of IgE recognising individual Can f 6 epitopes including those predicted in this study (Fig. 5A,B). Notably, the reduction in serological reactivity of wild-type and mutated rCan f 6 proteins was differentially assessed depending on whether the structured (ELISA-based inhibition experiments) or denatured (western blotting under an SDS-PAGE condition) allergen was used for the assays. Recently, Wang *et al*.^24^ determined five possible Can f 6 epitopes by peptide-based epitope mapping, and suggested that these epitopes were sequential epitopes due to the consecutive amino acids. Notably, one of the epitopes they determined (SDIKEKIEENGS) encompasses the mutated sites of both Can f 6-mu-2 (KEK) and mu-3(EEN). The fact that IgE reactivity of denatured rCan f 6-mu-2 was diminished compared with rCan f 6 regardless of serum (Fig. 5C), is consistent with the notion that it is a sequential epitope. However, this mutation had no effect on the binding of IgE to structured rCan f 6 when sera from patients no. 25 and no. 35 were used (Fig. 5B). Thus, it is conceivable that IgE-Can f 6 binding is highly structure-dependent and cannot be assessed accurately when synthetic peptides are used instead of the structured protein.

Overall, our results indicate that the three possible epitopes predicted in this study are at least partly involved in IgE binding. However, there is a possibility that IgE selectively recognising Can f 6 or Fel d 4 also exists. Therefore, further studies may be needed for comprehensive mapping of Can f 6 epitopes.

## Materials and Methods

### Specimen collection and ethical statement

Studies with human samples were conducted under the approval by the Ethics Review Committee of Sagamihara National Hospital. All experiments were performed in accordance with the ethical guidelines laid down in the Helsinki Declaration by the World Medical Association. The sera used in this study were collected from 38 consecutive outpatients with dog allergies, assessed by the skin prick test, who visited Sagamihara National Hospital, Japan (Table S1). Sera from six allergic donors without dog and/or cat allergies were also collected as controls (Table S1). Written informed consent was obtained from all patients.

### Preparation of the rCan f 6 and rFel d 4 expression plasmids

The nucleotide sequence of Can f 6 and Fel d 4 cDNAs was obtained from GenBank (accession numbers HE653774 and AY497902). Using Signal IP4.1 software (http://www.cbs.dtu.dk/services/SignalP), 597-nucleotide (nt) open reading frame (ORF) was found in the cDNA and the first 69-nt sequence of the ORF was predicted to encode a 23-amino acid (aa) signal peptide. Thus, the region encoding a putative and mature Can f 6 protein was amplified by PCR using the primer sets listed in Table S2. The PCR products were digested with *Bam*HI and *Sal*I restriction enzymes and inserted into the expression plasmid pGEX4T-2, which produces rCan f 6 harbouring an additional 2 amino acid residues (Gly-Ser) at its N-terminus. After sequence validation of the cloned PCR product, BL21 (DE3) *Escherichia coli* cells were then transformed with the plasmid construct pGEX4T-2-rCan f 6.

### Site-directed mutagenesis of Can f 6

Can f 6 was mutated at select residues to generate three mutated proteins, rCan f 6-mu-1, mu-2, and mu-3. Mutagenesis was performed using mutation-specific oligonucleotide primers containing each mutation (Table S2). Can f 6 internal amino acid substitution was performed by a megaprimer PCR^25^. The PCR products were digested with *Bam*HI and *Sal*I restriction enzymes and subcloned into the pGEX 4T-2 vector to generate mutated rCan f 6 proteins.

### Expression and purification of rCan f 6 and rFel d 4

To express the recombinant Glutathione S-transferase (GST)-fused rCan f 6 and rFel d 4, *E. coli* cells harbouring pGEX-4T-2-rCan f 6 were cultured at 37 °C for 16 h. *E. coli* cells were harvested by centrifugation (8,000 rpm for 5 min at 4 °C) and disrupted by ultrasonication followed by centrifugation. The supernatant was filtered using 0.45-µm disk filters and loaded onto a Glutathione Sepharose 4B column (GE Healthcare, Little Chalfont, UK). The column was washed with 1% Triton X-100 in PBS followed by PBS alone, after which thrombin (Sigma-Aldrich, St. Louis, MI, USA) was loaded onto the column and incubated at room temperature overnight to cleave rCan f 6 and rFel d 4 from GST. rCan f 6 and rFel d 4 were eluted by loading PBS onto the column and then subjected to further purification by gel filtration chromatography using HiLoad 16/600 Superdex 75 pg (GE Healthcare) with PBS. Protein concentration was determined by measuring the absorbance at 280 nm. Mutated rCan f 6 proteins were expressed and purified as described for rCan f 6.

### Mass spectrometry

The molecular mass of rCan f 6 proteins was determined by the AXIMA confidence spectrometer (Shimadzu Co., Kyoto, Japan) in the positive ion mode. rCan f 6 matrix assisted laser desorption/ionization-time of flight (MALDI-TOF) mass spectrometry was carried out in linear mode using sinapinic acid (Tokyo Chemical Industry Co. Ltd., Tokyo, Japan) as matrices. The lyophilised sample (0.1–0.5 nmol) was dissolved in 0.05% trifluoroacetic acid (TFA)/50% CH_3_CN (1 µL) mixed with 2 µL of a matrix solution (10 mg/mL) and then air-dried on the sample plate.

### Analytical ultracentrifugation-sedimentation velocity (AUC-SV)

AUC-SV experiments were performed using the ProteomeLab XL-I Analytical Ultracentrifuge (Beckman-Coulter, Fullerton, CA, USA). Sample concentration was adjusted to 50 µM. Runs were carried out at 60,000 rpm with a temperature of 20 °C using aluminium double-sector centrepieces and a four-hole An-60 Ti analytical rotor equilibrated to 20 °C. Sedimentation behaviour was monitored with UV detection optics at 280 nm. All SV raw data were analysed by the continuous *C*(s) distribution model using the SEDFIT 14.4 software^26^. We used a resolution of 500 increments between 0 and 15S as well as maximum entropy regularization (p = 0.68).

### Crystallisation and X-ray data collection

All crystallisation experiments were performed at 20 °C using the sitting-drop method of vapour diffusion. Under optimised conditions, a crystallisation drop containing 2 µL rCan f 6 protein solution (17 mg/mL in 20 mM phosphate buffer, pH 7.0) and 2 µL precipitant solution (1.0 M ammonium dihydrogen phosphate and 20% [w/v] PEG3350) was equilibrated against 200 µL precipitant solution. Crystals suitable for X-ray diffraction appeared within one week in crystallisation drops (Supplementary Fig. S8). For data collection, crystals were transferred directly from their mother liquor to a nitrogen cold stream at −173 °C. Diffraction data were collected from beam-line BL38B1 (λ = 1.0000 Å) at SPring-8 (Hyogo, Japan) using an MX225-HE detector (Rayonix LLC, Evanston, Illinois, USA) at a cryogenic temperature (−173 °C). Data were integrated and scaled using HKL2000 software^27^. Processing statistics are summarised in Table 1.

### Structure determination and refinement

The structure was solved by molecular replacement using MOLREP^28^ in the CCP4i package^29^ with the structure of a salivary lipocalin from boar (Protein Data Bank ID: 1GM6) as the search model; this template shares 61% sequence identity with Can f 6. Although four molecules of Can f 6 were found in the asymmetric unit, non-crystallographic symmetry restraints were not applied during the model refinement. Iterative rounds of model building and refinement were carried out using COOT^30^ and Refmac5^31^ in the CCP4i package, respectively. Graphical representations were prepared using PyMOL software (http://www.pymol.org). Refinement statistics are summarised in Table 1.

### IgE enzyme-linked immunosorbent assay (ELISA)

rCan f 6 and mutants were immobilised onto ELISA plates (IWAKI, Tokyo, Japan) overnight at 4 °C. After washing with PBS, the plate wells were blocked with 3% skim milk in PBS for 1 h at room temperature. Sera from 38 dog-allergic patients and 6 non-dog-allergic donors were diluted 1:250 with PBS, added to the wells (100 µL/well), and incubated for 1 h at 37 °C. Subsequently, biotin-labelled goat anti-human IgE antibody (0.5 mg/mL; Milford, MA, USA) diluted 1:5,000 with PBS was added to the wells (100 µL/well) and incubated for 1 h at 37 °C. Next, Pierce^®^ High Sensitivity Streptavidin-HRP (1.1 mg/mL; Thermo Fischer Scientific, Waltham, MA, USA) diluted 1:10,000 times with PBS was added (100 µL/well). For detection of allergen-IgE complexes, TMB Substrate Reagent (BD Biosciences, Bedford, MA, USA) was added (100 µL/well) and incubated for 15 min at room temperature. To stop the reaction, 1 N H_2_SO_4_ was used (100 µL/well). Absorbance at 450 nm was measured using the BioTek^TM^ Eon^TM^ Microplate Spectrophotometer (BioTek Instruments Inc, Winooski, VT, USA).

### Western blotting

Western blotting was performed as described previously^32^. rCan f 6 proteins were separated by 12% native polyacrylamide gel electrophoresis (PAGE) and 15% SDS-PAGE, transferred to 0.2-µm polyvinylidene fluoride (PVDF) membranes (Merck Millipore, Billerica, MA, USA), and then stained with Ponceau S (Tokyo Chemical Industry, Tokyo, Japan). After blocking for 1 h at room temperature in 3% skim milk, the membrane was incubated with diluted (1: 100) sera from dog-allergic patients for 16 h at 4 °C. After washing with Tris-buffered saline/Tween 20 (TBST), the blots were incubated with a biotin-labelled antibody against human IgE (SeraCare) and HRP-conjugated streptavidin (Thermo Fisher Scientific). Immunoreactivity was visualised using the Immobilon Western Chemiluminescent HRP Substrate (Merck Millipore) and a LAS4000 imaging device (GE Healthcare).

## Supplementary information

Supplementary figures and tables

## Data Availability

Atomic coordinates of rCan f 6 have been deposited in the Protein Data Bank under the file name 5 × 7Y.
