# Supplementary material for: Crystal structure of the dog allergen Can f 6 and structure-based implications of its cross-reactivity with the cat allergen Fel d 4
Source: Sci Rep. 2019 Feb 6;9:1503. doi: 10.1038/s41598-018-38134-w (PMC6365566; doi:10.1038/s41598-018-38134-w)
Supplement: Supplementary file 1 — Supplementary figures and tables [file 41598_2018_38134_MOESM1_ESM.pdf]

Crystal structure of the dog allergen Can f 6 and structure-based implications of its cross-reactivity with the cat allergen Fel d 4

Kenji Yamamoto[1,*], Osamu Ishibashi[1,*], Keisuke Sugiura[1], Miki Ubatani[1], Masaya Sakaguchi[1], Masatoshi Nakatsuji[1], Shigeru Shimamoto[2], Masanori Noda[3], Susumu Uchiyama[3], Yuma Fukutomi[4], Shigenori Nishimura[1] & Takashi Inui[1]

[1]Department of Applied Life Sciences, Graduate School of Life and Environmental Sciences, Osaka Prefecture University, 1-1 Gakuen-cho, Naka-ku, Sakai 599-8531, Japan; [2]Faculty of science and engineering, Kinki University, 3-4-1 Kowakae, Higashi-Osaka 577-8502, Japan; [3]Department of Biotechnology, Graduate School of Engineering, Osaka University, 2-1 Yamadaoka, Suita 565-0871, Japan; [4]Clinical Research Center for Allergy and Rheumatology, Sagamihara National Hospital, 18-1 Sakuradai, Minami-ku, Sagamihara 252-0392, Japan

Correspondence and requests for materials should be addressed to T.I. (email: inuit@bioinfo.osakafu-u.ac.jp)

* Equally contributed to this work.

Supplementary Table S1 Serological data of the sera used in this study

Subject No.	Age	Gender	sIgE(kUA/ml) dog dander	sIgE(kUA/ml) cat dander	Subject No.	Age	Gender	sIgE(kUA/ml) dog dander	sIgE(kUA/ml) cat dander
**1**	29	F	42.60	n.d.	**23**	23	M	67.50	10.5
**2**	63	F	70.10	n.d.	**24**	21	F	55.60	17.8
**3**	39	M	18.90	0.65	**25**	52	F	>100	43.9
**4**	38	F	23.00	1.02	**26**	32	M	5.17	84.3
**5**	18	F	77.30	n.d.	**27**	52	M	10.70	0.65
**6**	34	F	40.70	n.d.	**28**	57	M	41.70	8.62
**7**	32	M	24.40	n.d.	**29**	60	M	46.30	0.52
**8**	48	F	10.30	n.d.	**30**	26	F	58.90	13.6
**9**	40	M	15.20	n.d.	**31**	38	M	14.10	33.1
**10**	41	F	12.80	n.d.	**32**	29	F	6.62	13.0
**11**	28	F	78.80	n.d.	**33**	23	F	8.04	12.6
**12**	26	F	14.90	72.9	**34**	24	F	4.49	71.9
**13**	39	F	14.20	20.4	**35**	39	F	>100	12.5
**14**	56	F	70.60	n.d.	**36**	30	F	23.70	3.14
**15**	23	F	>100	n.d.	**37**	30	F	>100	2.43
**16**	22	F	>100	n.d.	**38**	58	F	58.10	9.33
**17**	40	F	>100	n.d.	**N1**	37	F	<0.35	<0.35
**18**	27	F	>100	n.d.	**N2**	27	M	<0.35	<0.35
**19**	26	F	>100	n.d.	**N3**	42	F	<0.35	<0.35
**20**	22	F	4.16	50.3	**N4**	51	F	<0.35	<0.35
**21**	20	M	7.34	14.7	**N5**	62	F	<0.35	<0.35
**22**	30	F	91.30	2.75	**N6**	40	F	<0.35	<0.35

n.d.: not determined

Supplementary Table S2 Primers used in this study

**Can f 6 forward**	5’-GGGGGATCCCACGAGGAAGAAAAC-3’
**Can f 6 reverse**	5’-TTTGTCGACTCACTCAGCACTGGAGAC-3’
**mu-1**	5’-GGAAACTTCGATATTGCAGCGGCTTCGGGAGATTGGTAT-3’
**mu-2**	5’-TTGGCCTCAGATATCGCGGCAGCGATAGAAGAAAATGGC-3’
**mu-3**	5’-ATCAAGGAAAAGATAGCAGCAGCTGGCAGCATGAGGGTT-3’

Underlines denote the nucleotides where mutations were introduced

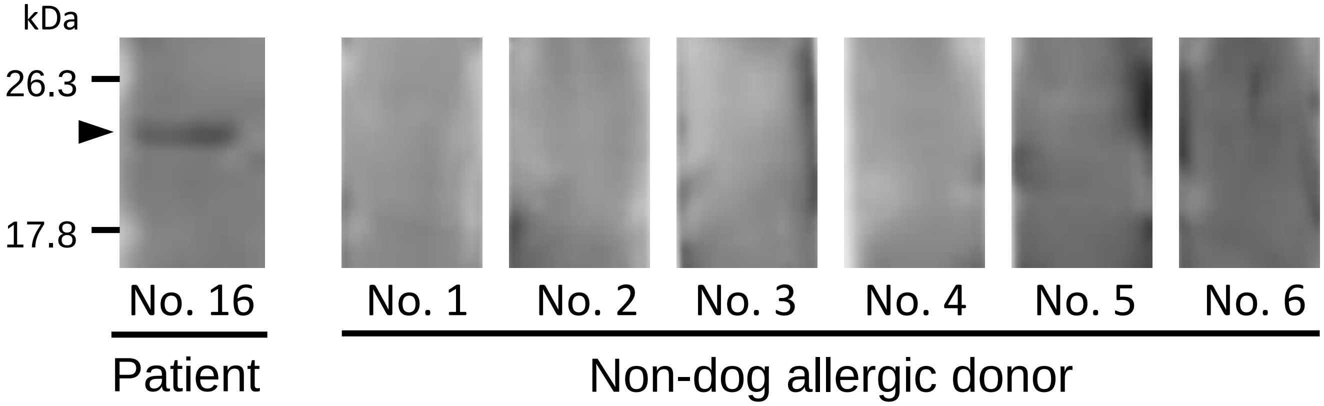

Supplementary Fig. S1 Detection of Can f 6-reactive IgE by western blotting. Sera from patient 16 and six non-dog-allergic donors were subjected to western blotting for Can f 6-reactive IgE. The arrowhead indicates the position of rCan f 6.

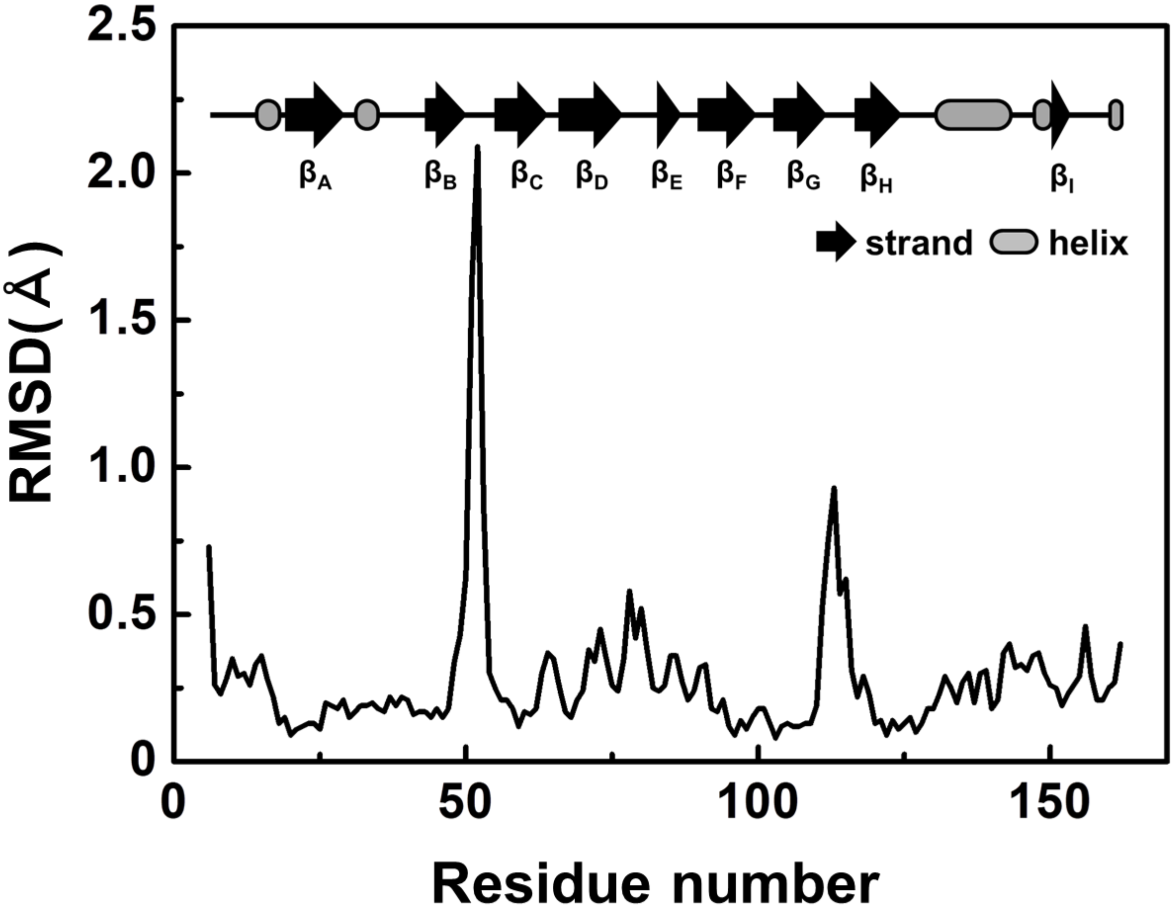

Supplementary Fig. S2 Average root mean square deviation (RMSD) of main-chain atoms from the mean structure averaged over the four molecules in the asymmetric unit as a function of residue number. Positions of typical secondary structures in rCan f 6 (chain A) are also shown.

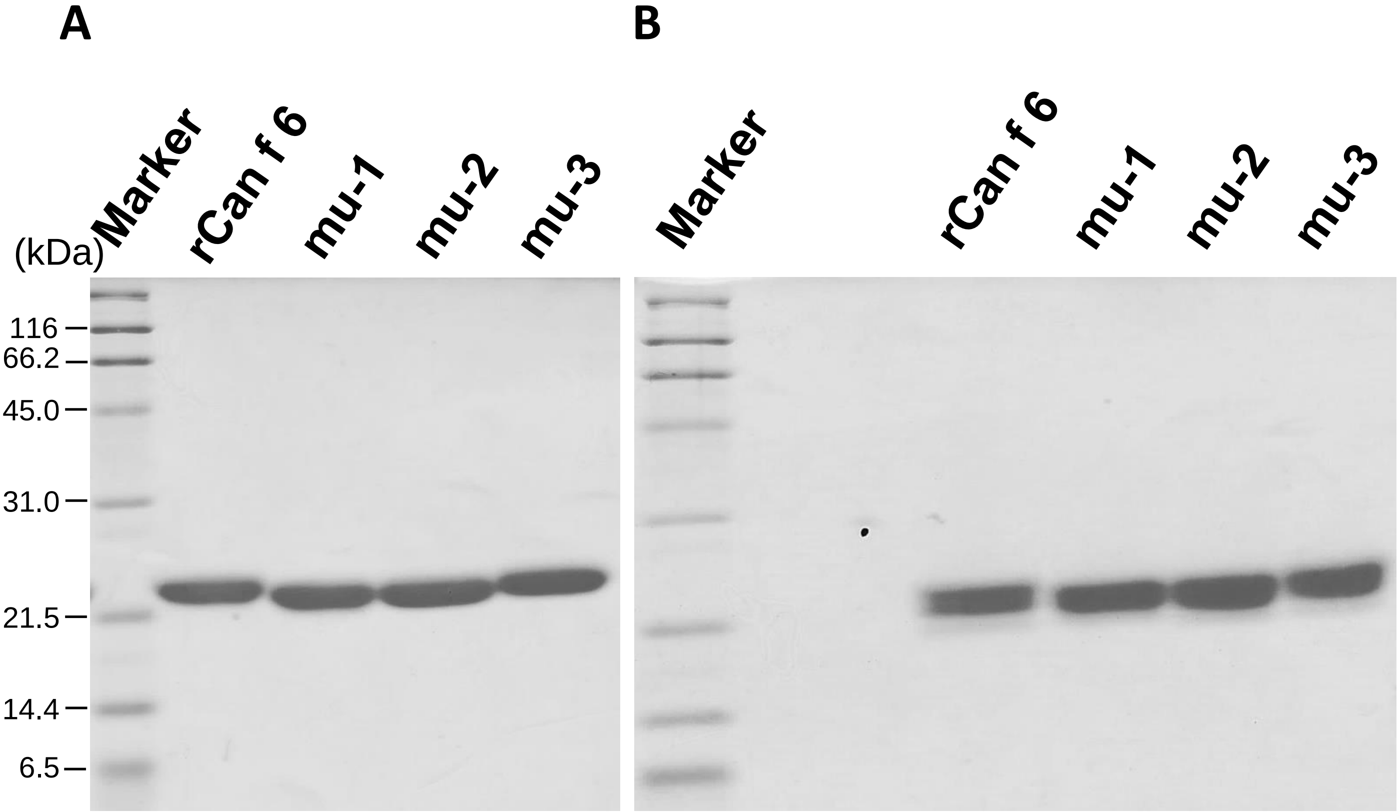

Supplementary Fig. S3 SDS-PAGE analyses of the purified mutated rCan f 6 proteins. Purified recombinant proteins of mutated and non-mutated rCan f 6 (2 µg/lane) were electrophoretically separated under (A) reducing or (B) non-reducing conditions and stained with Coomassie Brilliant blue.

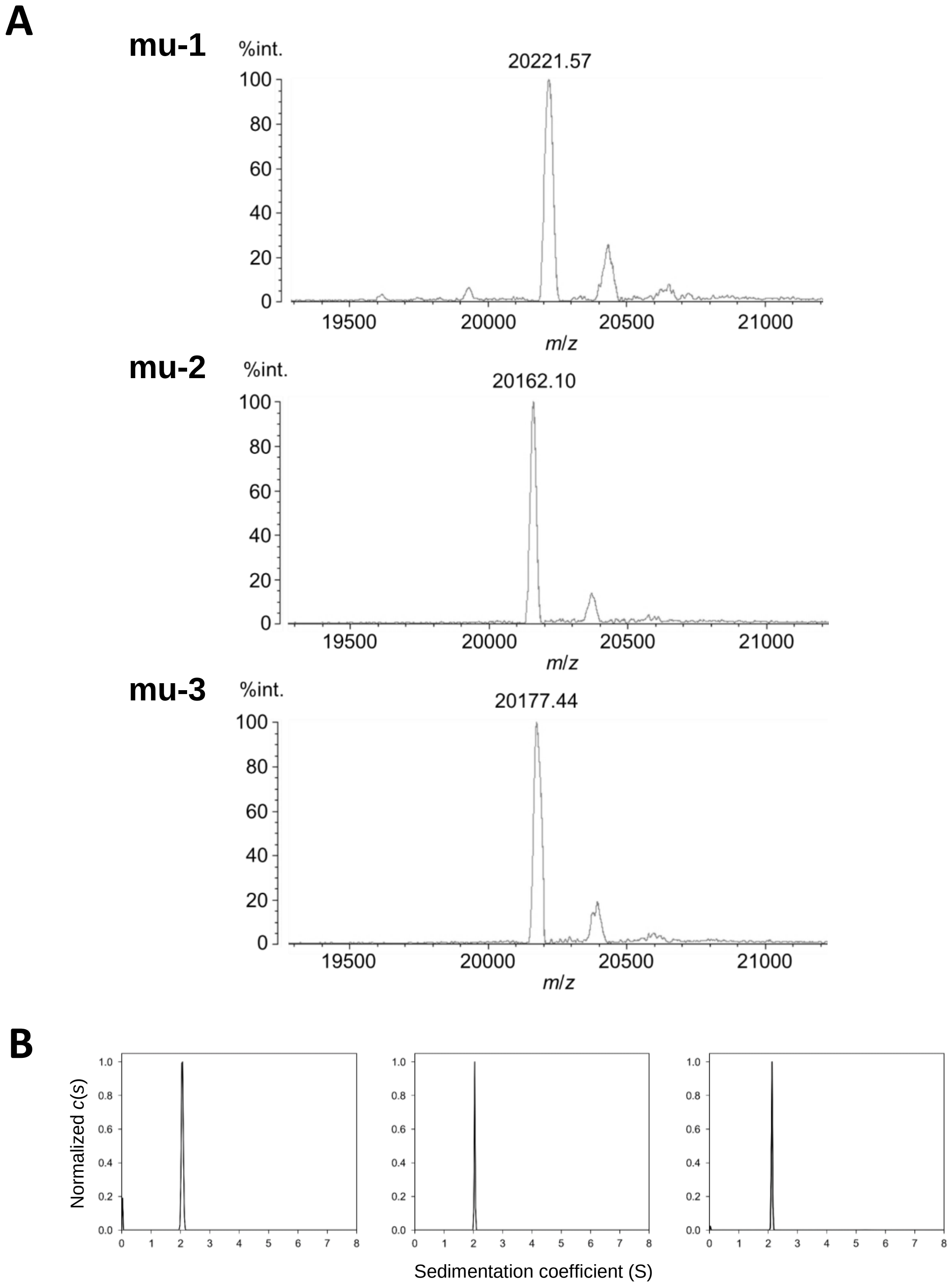

Supplementary Fig. S4 Molecular mass determination of the mutated rCan f 6 proteins. (A) MALDI-TOF mass spectra of the mutated rCan f 6 proteins. The *m/z* values of the main peaks correspond to the deduced molecular mass of individual recombinant proteins (mu-1: 20221.77, mu-2: 20164.72, mu-3: 20177.85). (B) Distribution states of the mutated rCan f 6 proteins analysed by AUC-SV. The molecular mass of rCan f 6-mu-1, mu-2, and mu-3 were calculated as 18.7, 19.1, and 17.7 kDa, respectively.

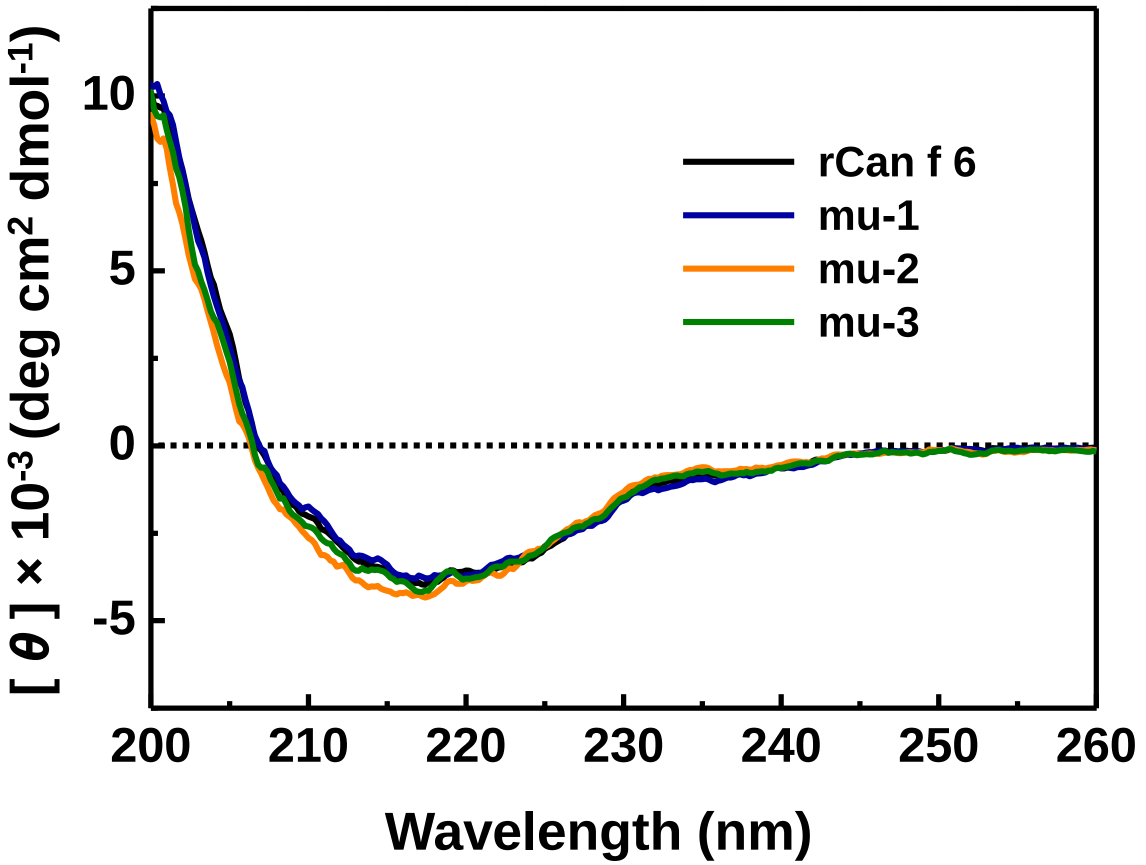

Supplementary Fig. S5 Circular dichroism (CD) spectra of non-mutated and mutated rCan f 6 proteins. CD measurements were performed with a far-UV range at 200–260 nm with a J-820 spectropolarimeter (Jasco, Tokyo, Japan). The temperature of the sample solution in the cuvette was controlled at 37° C by a Peltier PTC-423 L thermo-unit (Jasco). The path length of the optical quartz cuvette was 1.0 mm and the sample concentration for the far-UV range was 5 μM in PBS (pH 7.4). The data are expressed as the molar residue ellipticity [*θ*].

Supplementary Fig. S5 Yamamoto, K. *et al.*

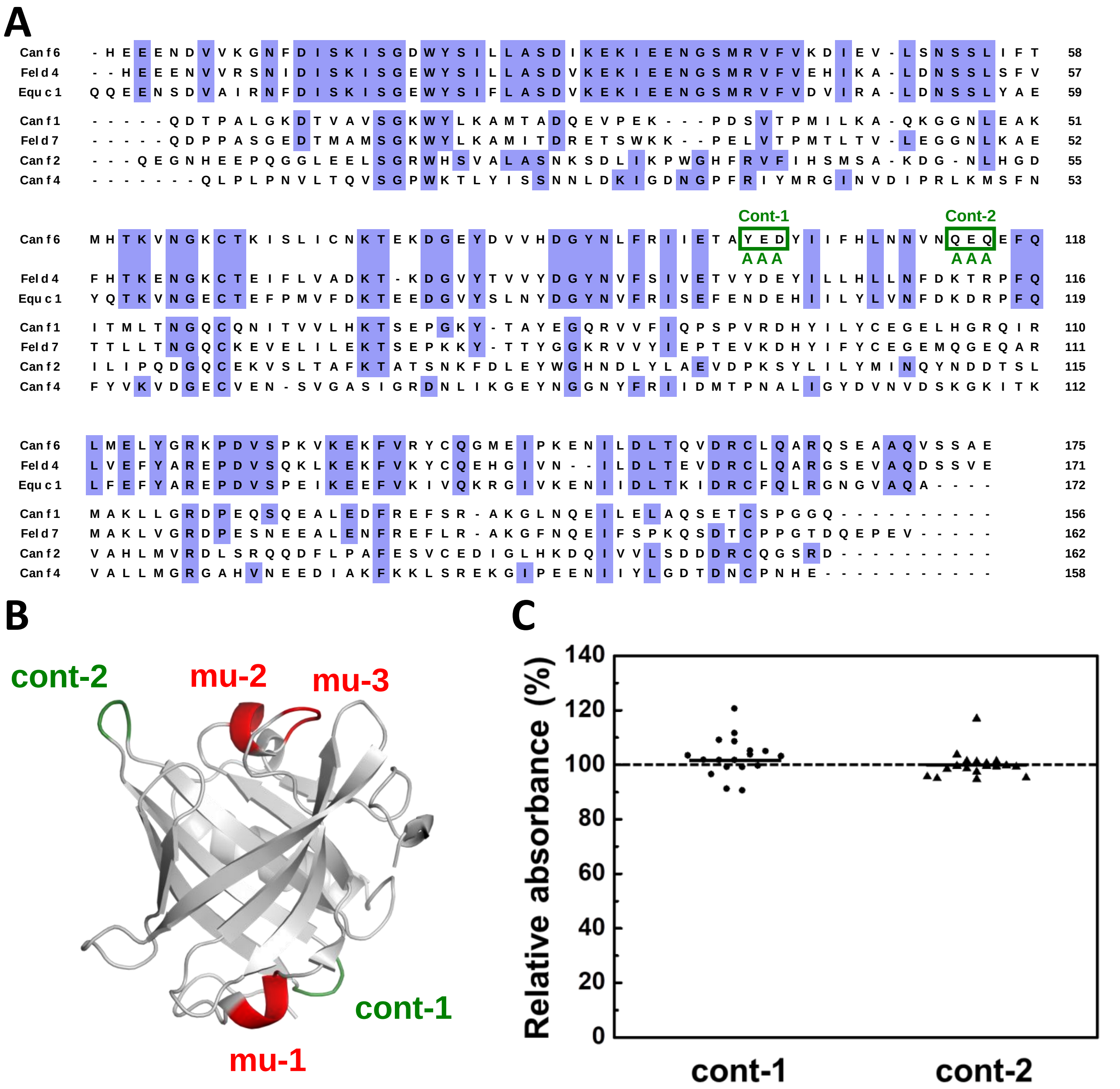

Supplementary Fig. S6 Can f 6 mutations within surface-facing regions that are less conserved among the lipocalin allergens and their effect on IgE reactivity. (A) Three successive amino acids at two sites within the less conserved region were substituted with triple alanine and designated rCan f 6-cont-1 and cont-2. (B) Schematic representation of the mutation sites of rCan f 6-cont proteins (shown in green) as well as those of rCan f 6-mu proteins. (C) Relative IgE reactivity to rCan f 6-cont proteins compared with rCan f 6 was evaluated by ELISA. Can f 6-reactive sera from 18 patients were subjected to this assay. Lines in individual columns denote medians.

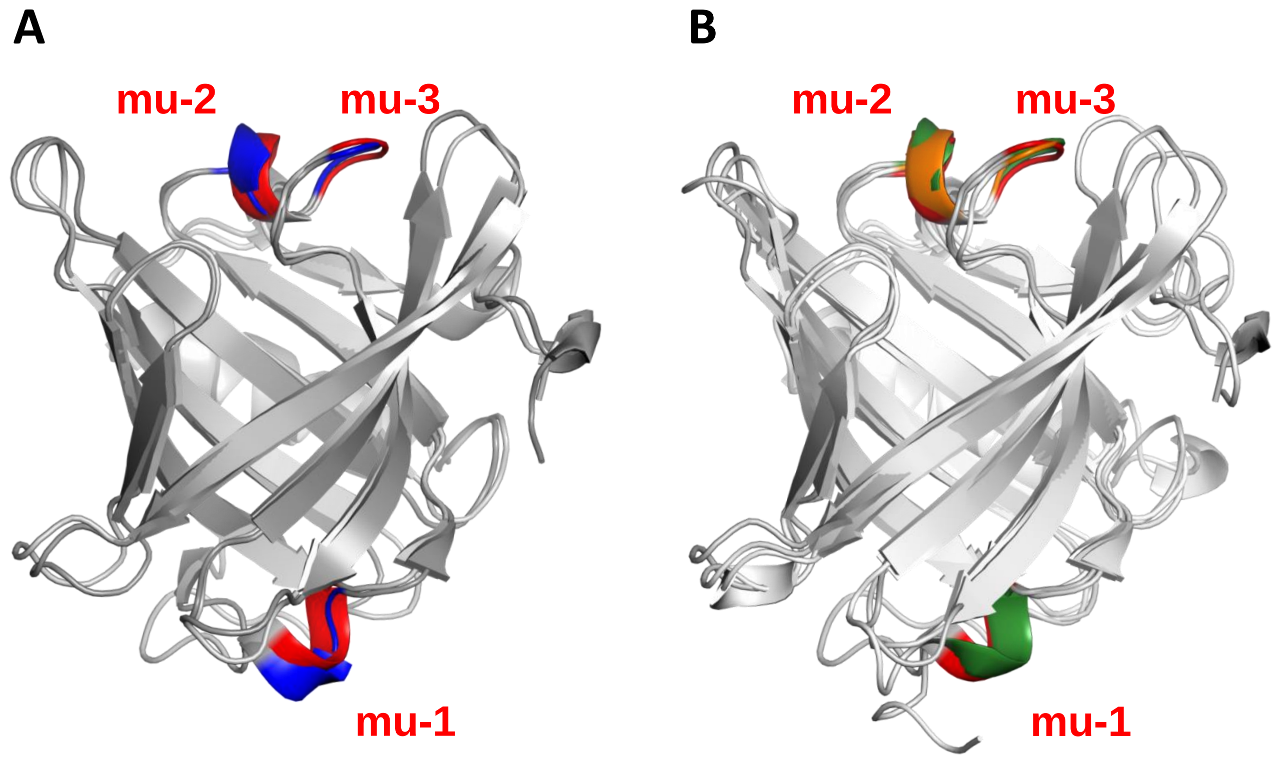

Supplementary Fig. S7 Structural Similarities of Can f 6 to Equ c 1, Can f 2, and Can f 4. Superimposed X-ray structures (A) between Can f 6 and Equ c 1 and (B) among Can f 2, Can f 4 and Can f 6 are shown. The mutation sites of rCan f 6-mu-1, mu-2, and mu-3 are indicated in red and the corresponding sites of Equ c 1, Can f 2, and Can f 4 are indicated in blue, green, and orange, respectively.

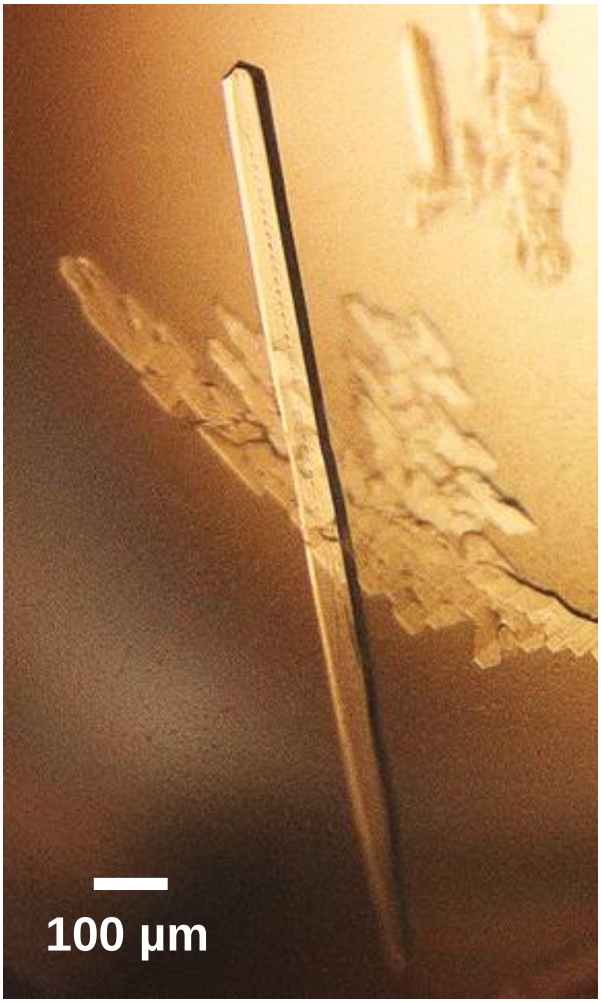

Supplementary Fig. S8 Images of the representative protein crystals of rCan f 6.
